# Unravelling the fatty acid profiles of different polychaete species cultured under integrated multi-trophic aquaculture (IMTA)

**DOI:** 10.1038/s41598-021-90185-8

**Published:** 2021-05-24

**Authors:** Daniel Jerónimo, Ana Isabel Lillebø, Elisabete Maciel, M. Rosário M. Domingues, Javier Cremades, Ricardo Calado

**Affiliations:** 1 ECOMARE & CESAM & Departamento de Biologia, Universidade de Aveiro, Campus Universitário de Santiago, 3810-193 Aveiro, Portugal; 2 ECOMARE & CESAM & Departamento de Química, Universidade de Aveiro, Campus Universitário de Santiago, 3810-193 Aveiro, Portugal; 3 Centro de Espectrometria de Massa, LAQV REQUIMTE, Departamento de Química, Universidade de Aveiro, Campus Universitário de Santiago, 3810-193 Aveiro, Portugal; 4 Coastal Biology Research Group (BioCost), Facultad de Ciencias & CICA, Universidade da Coruña, 15071 A Coruña, Spain

**Keywords:** Sustainability, Marine biology

## Abstract

Polychaetes can be successfully employed to recover otherwise wasted nutrients present in particulate organic matter (POM) of aquaculture effluents. The present study describes the fatty acid (FA) profile of four different polychaete species cultured in sand filters supplied with effluent water from a marine fish farm. The FA profile of cultured and wild *Hediste diversicolor* was compared and revealed a ≈ 24.2% dissimilarity, with cultured biomass displaying a higher content in two essential *n*-3 highly unsaturated FA (HUFA) (EPA [20:5 *n*-3] and DHA [22:6 *n*-3]—eicosapentaenoic and docosahexaenoic acid, respectively). The comparison of the FA profile of cultured *H. diversicolor* with that of other polychaete species whose larvae successfully settled on the sand filters (*Diopatra neapolitana*, *Sabella* cf. *pavonina* and *Terebella lapidaria*) revealed that their FA profile, which is here described for the first time, displayed high levels of EPA and DHA (≈ 1.5–4.8 and 1.0–1.1 µg mg^−1^ DW, respectively). The highest concentration of total FA per biomass of polychaete was recorded in *H. diversicolor* and *T. lapidaria*, with both species being the ones whose FA profiles revealed a lowest level of dissimilarity and more closely resembled that of the aquafeed used in the fish farm. In the present work it was demonstrated that it is possible to produce polychaetes biomass with high nutritional value through an eco-design concept such as integrated multi-trophic aquaculture (IMTA). Indeed, this framework promotes a cleaner production and, in this specific case, allowed to recover essential fatty acids that are commonly wasted in aquaculture effluents.

## Introduction

Aquaculture has grown globally 5.8% per year during the period 2001–2016 and continues to grow faster than any other food production sector^1^. In 2016, this industry produced 110.1 million tonnes of food fish and aquatic plants with an estimated value of USD 243.3 billion^1^. It is through the growth and development of this industry that can be possible to supplement human needs in *n*-3 highly unsaturated fatty acids (HUFA). A dose of 500 mg/day of eicosapentaenoic (20:5 *n*-3 [EPA]) and docosahexaenoic (22:6 *n*-3 [DHA]), *n*-3 HUFA, is recommended to reduce the risk of cardiovascular disease^2–5^. Based on this recommended dose to maintain a good cardiac wellness, there is a global requirement of approximately 0.4 million metric tonnes of *n*-3 HUFA per year^5^. Our needs in these essential fatty acids (EFA) are due to limitations that vertebrate species exhibit in the de novo synthesis of these molecules due to the lack of desaturases (Δ12 and Δ15) responsible to produce polyunsaturated fatty acids (PUFA) from oleic acid (18:1 *n*-9), thereby making their inclusion in the aquafeeds essential^5–7^. Marine fish for example incorporate in their tissues with little or no modification the fatty acids (FA) from lower trophic levels and, as such, some species may present well-defined FA signatures depending on their diet^6^. These EFA are included in formulated aquafeeds to satisfy the needs of cultured species, but especially so that these at the end of a productive cycle exhibit an optimal profile for human nutrition^5^. Presently, balanced aquafeeds are formulated using fish meal and fish oil (mainly for marine finfish and shrimp), two increasingly scarcer and costly marine based resources^1^. Their inclusion has been optimized over time and today´s formulas contain less than 10% and 20% of their protein and oil-based composition derived from these sources, respectively^8, 9^. Nonetheless, to sustain the expected global growth of aquaculture the search for new sources of EFA is of utmost importance.

Polychaete species can play a key-role on this quest for new sources of valuable EFA. These species can uptake nutrients present in aquaculture effluents in the form of particulate organic matter (POM) and, therefore, their culture under integrated multi-trophic aquaculture (IMTA) conditions has gained a growing attention. In marine IMTA systems, extractive organisms act at different trophic levels targeting the recovery of particulate organic matter (POM deposit feeders such as detritivores fish or invertebrates), dissolved organic matter (DOM filter feeders such as invertebrates) and dissolved inorganic nutrients (primary producers such as micro or macroalgae and halophytes)^10–17^. This concept enables POM-extractive organisms to incorporate otherwise wasted *n*-3 HUFA contained in the uneaten fraction of aquafeeds supplied to farmed species^18–20^. Indeed, as POM deposit feeders, polychaetes can play an important role in the recovery of these EFA (e.g., EPA [20:5 *n*-3] and DHA [22:6 *n*-3]). Species such as *Hediste diversicolor*^6, 15, 21–28^, *Perinereis nuntia* and *P. helleri*^29^, *Nereis virens*^30^, *Abarenicolla pusilla*^31^, *Sabella spallanzanii*^32^ and *Arenicola marina*^27^ have already been tested as IMTA extractive organisms. The ragworm *H. diversicolor* in particular revealed a significantly ability to retain valuable HUFA (such as EPA [20:5 *n*-3] and DHA [22:6 *n*-3]) from uneaten fish feeds that would otherwise be lost to the environment and negatively impact adjacent aquatic ecosystems^6, 23^. Some polychaete species evidenced de novo EFA biosynthesize, while their fat content also reflected the fat content of the diet^33^. These organisms are already known to play a central dietary role on the nutrition and production of some fish and crustacean species (e.g., soles, shrimps and crabs), being often used to trigger gonad maturation and spawning^17, 34, 35^. The development of production models that include polychaete species appears as an opportunity to meet the growing demand for these worm’s biomass. The potential market to produce for example *H. diversicolor* in polychaete assisted sand filters (PASF) under IMTA conditions (final productivities: 7000 ind. m^−2^ – 2300 g fresh weight biomass) was evaluated in approximately 90 € m^−2^ (if sold as bait)^15^. Unfortunately, there is no reference value available that may allow us to estimate what would be the tentative price of this DHA-rich polychaetes biomass if it was to be sold frozen (or dehydrated) and free of pathogens for premium aquafeeds formulation (e.g., finishing and breeding diets). The values of global harvest of polychaetes in 2016 (approx. 121,000 tonnes) are comparable to many of the world´s most important fisheries^36^. It has also already been acknowledged that their collection from the wild is likely insufficient to satisfy the global market demands (either as bait for sports fishing or as feed for aquaculture) and that this practice drives a multitude of negative environmental impacts^37^. Multiple objectives were target with the development of polychaete production models, such as the reduction of indiscriminate harvesting, reduction of imports of non-native species, development of new aquaculture products and the unravelling of new market and products^17, 38, 39^.

The present study evaluated the valorisation potential of several polychaete species produced through IMTA, a concept which promotes a cleaner production, as otherwise wasted nutrients can be converted into valuable polychaete biomass. This eco-design concept maximizes and diversifies production and increases efficiency in the use of nutrients, water and energy. Therefore, the first objective of the present study was to identify the FA profile of *H. diversicolor* stocked in tanks with a sand bed being supplied with an organic rich effluent from earthen ponds used for semi-intensive finfish grow-out and compare it with the FA profile of wild conspecifics. The FA profiles of *H. diversicolor* stocked in the tanks was also compared with that of the most representative polychaete species whose planktonic larvae successfully settled on the sand beds, namely *Diopatra neapolitana*, *Sabella* cf. *pavonina* and *Terebella lapidaria*. Finally, the FA profiles of cultured polychaetes were compared to that of the formulated aquafeed provided to the finfish in earthen ponds, in order to identify which species mimicked more closely the FA profile of the aquafeed, hence holding a greater potential to be more readily incorporated in its formulation.

## Results

### Comparison of fatty acid profiles of wild and IMTA-cultured Hediste diversicolor

**Figure 1:**
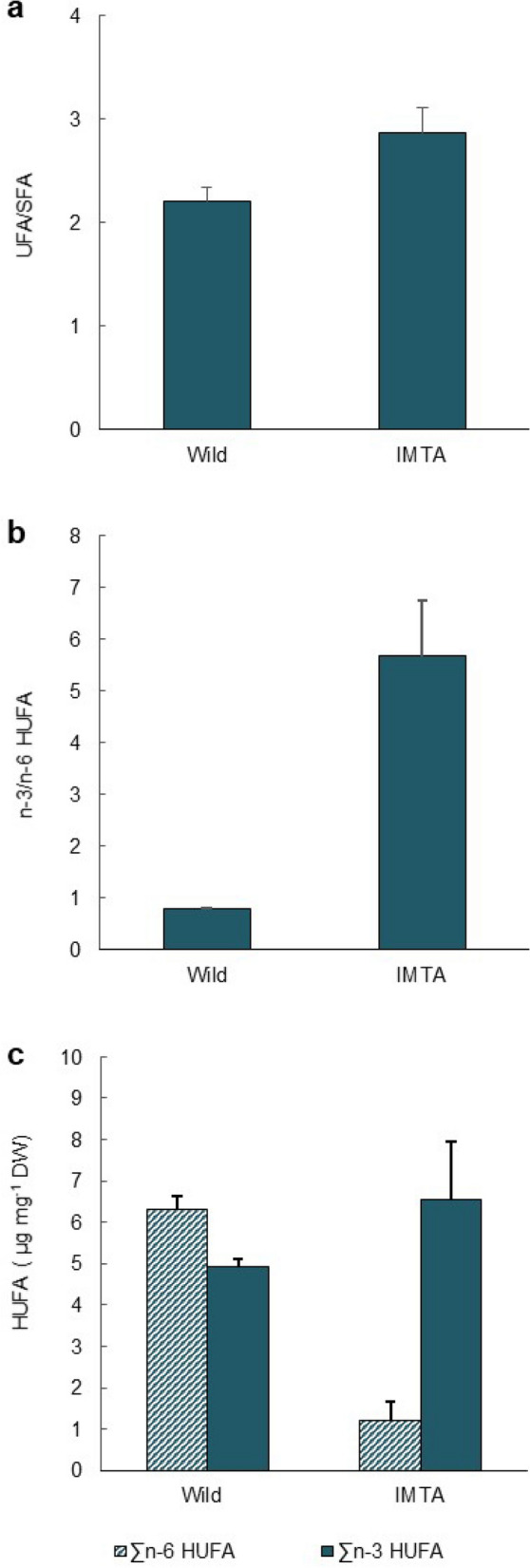
Fatty acid profile of wild and IMTA-cultured *Hediste diversicolor*: (**a**) unsaturated and saturated fatty acids ratio (UFA/SFA); (**b**) *n*-3/*n*-6 highly unsaturated fatty acids ratio (*n*-3/*n*-6 HUFA); (**c**) sum of *n*-3 and *n*-6 highly unsaturated fatty acids content (∑n-3 and n-6 HUFA; values in µg mg^−1^ DW). Average values ± SD (n = 5).

The FA profiles of wild and IMTA-cultured *H. diversicolor* are detailed in Table 1 (FA from microbiome and iso and anteiso are presented in Supplementary Table S1). Significant differences were found between the FA profiles (ANOSIM test; R = 1; *p* = 0.008), with the SIMPER analysis 50% cut-off (Table 2) revealing an average dissimilarity of 24.2%. The higher content of alpha-linolenic (18:3 *n*-3 [ALA]), arachidonic (20:4 *n*-6 [ARA]) and adrenic (22:4 *n*-6 [AdA]) acids recorded in wild polychaetes biomass contributed greatly for these differences, as well as the higher content of linoleic acid (18:2 *n*-6 [LA]) and DHA (22:6 *n*-3) recorded in IMTA-cultured specimens. The 7,10,13-hexadecatrienoic acid (16:3 *n*-3) and gamma-linolenic acid (18:3 *n*-6) were identified only in wild polychaetes biomass, while DHA (22:6 *n*-3) was only identified in cultured polychaetes biomass. In general, IMTA-cultured polychaetes exhibited a FA profile with a higher unsaturated/saturated FA (UFA/SFA) ratio (Fig. 1a). By analysing the HUFA profile, it was also possible to verify that IMTA-cultured polychaetes exhibited a higher *n*-3/*n*-6 HUFA ratio, featuring an increment of *n*-3 HUFA (including EPA and DHA) and a reduction of *n*-6 HUFA (Fig. 1b,c, respectively).

**Table 1:** Fatty acid composition (µg mg^−1^ DW) of wild and IMTA-cultured polychaete species and aquafeed added to fish.

Fatty acid	*Hediste diversicolor* (wild)	*Hediste diversicolor*	*Diopatra neapolitana*	*Sabella* cf. *pavonina*	*Terebella lapidaria*	Aquafeed
14:0	0.39 ± 0.20	0.85 ± 0.15	0.27 ± 0.08	1.19 ± 0.71	0.46 ± 0.08	1.30 ± 0.31
16:0	8.64 ± 0.71	6.70 ± 1.49	1.09 ± 0.22	4.31 ± 1.58	5.69 ± 0.48	16.78 ± 2.58
18:0	2.27 ± 0.17	1.86 ± 0.43	1.22 ± 0.17	2.55 ± 0.66	1.47 ± 0.15	6.47 ± 1.83
20:0	ND	ND	ND	ND	ND	0.28 ± 0.03
22:0	ND	ND	ND	ND	ND	0.14 ± 0.01
**∑SFA**	**11.30 ± 1.07**	**9.40 ± 2.04**	**2.58 ± 0.44**	**8.05 ± 2.93**	**7.62 ± 0.69**	**24.97 ± 4.25**
16:1 *n*-9	ND	ND	ND	ND	0.14 ± 0.03	3.50 ± 0.38
16:1 *n*-7	ND	ND	ND	ND	ND	0.24 ± 0.02
16:1 *n*-5	0.67 ± 0.11	1.70 ± 0.41	0.26 ± 0.04	1.18 ± 0.51	1.44 ± 0.25	ND
18:1 *n*-14	1.73 ± 0.09	1.33 ± 0.25	ND	ND	0.59 ± 0.03	ND
18:1 *n*-9 & n-7	3.76 ± 0.34	8.02 ± 2.43	0.67 ± 0.11	4.79 ± 0.83	7.22 ± 1.64	36.14 ± 3.66
20:1 *n*-13 & n-11	1.90 ± 0.07	2.29 ± 0.56	0.53 ± 0.09	0.21 ± 0.02	1.96 ± 0.28	ND
20:1 *n*-9	0.20 ± 0.11	0.05 ± 0.04	ND	0.61 ± 0.16	0.01 ± 0.01	2.33 ± 0.25
20:1 *n*-7	ND	ND	ND	ND	ND	0.18 ± 0.04
22:1	0.04 ± 0.01	0.24 ± 0.13	0.05 ± 0.02	0.04 ± 0.00	0.09 ± 0.03	2.25 ± 0.14
**∑MUFA**	**8.30 ± 0.48**	**13.63 ± 3.36**	**1.50 ± 0.20**	**6.82 ± 1.48**	**11.44 ± 2.11**	**43.86 ± 4.31**
16:3 *n*-3	0.07 ± 0.02	ND	ND	ND	ND	ND
18:2 *n*-6 (LA)	1.19 ± 0.10	3.78 ± 1.21	0.22 ± 0.04	0.71 ± 0.03	2.29 ± 0.61	16.72 ± 1.85
18:3 *n*-6	0.05 ± 0.01	ND	ND	0.08 ± 0.03	ND	0.10 ± 0.03
18:3 *n*-3 (ALA)	3.55 ± 0.27	0.63 ± 0.25	0.04 ± 0.01	0.11 ± 0.04	0.34 ± 0.08	2.80 ± 0.32
^Δ5,11^ 20:2 *n*-9	0.25 ± 0.03	0.53 ± 0.10	ND	ND	0.15 ± 0.03	ND
20:2 *n*-7	0.07 ± 0.01	0.07 ± 0.03	ND	ND	0.24 ± 0.02	ND
20:2 *n*-6	ND	ND	ND	ND	ND	0.49 ± 0.06
^Δ8,11^ 20:2 *n*-9	1.27 ± 0.06	1.11 ± 0.36	0.37 ± 0.06	0.23 ± 0.02	1.23 ± 0.26	ND
20:3 *n*-6	1.23 ± 0.61	0.58 ± 0.19	0.54 ± 0.28	0.78 ± 0.52	0.59 ± 0.18	ND
20:3 *n*-3	0.32 ± 0.02	0.10 ± 0.03	0.03 ± 0.01	0.05 ± 0.03	0.03 ± 0.01	0.15 ± 0.01
^Δ7,13^ 22:2 *n*-9	0.34 ± 0.06	0.72 ± 0.11	0.99 ± 0.22	ND	0.34 ± 0.05	ND
^Δ5,13^ 22:2 *n*-9	ND	ND	ND	1.50 ± 0.22	ND	ND
^Δ7,13,16^ 22:3	0.22 ± 0.08	0.22 ± 0.06	0.10 ± 0.03	ND	0.30 ± 0.03	ND
24:2C	ND	ND	ND	0.40 ± 0.07	ND	ND
**∑PUFA**	**8.58 ± 0.96**	**7.75 ± 1.85**	**2.30 ± 0.39**	**3.86 ± 0.79**	**5.52 ± 1.11**	**20.27 ± 2.24**
18:4 *n*-3	ND	ND	ND	ND	ND	0.58 ± 0.07
20:4 *n*-6 (ARA)	3.45 ± 0.07	0.65 ± 0.22	0.45 ± 0.07	0.71 ± 0.06	1.12 ± 0.11	0.39 ± 0.04
20:4 *n*-3	0.40 ± 0.05	0.09 ± 0.04	ND	0.03 ± 0.01	0.13 ± 0.03	0.29 ± 0.03
20:5 *n*-3 (EPA)	3.68 ± 0.13	4.83 ± 0.99	3.06 ± 0.36	1.46 ± 0.47	3.00 ± 0.32	2.11 ± 0.18
22:4 *n*-6 (AdA)	2.86 ± 0.25	0.55 ± 0.25	0.21 ± 0.04	0.04 ± 0.02	1.58 ± 0.08	ND
22:5 *n*-6	ND	ND	0.03 ± 0.02	0.02 ± 0.01	0.23 ± 0.03	ND
22:5 *n*-3 (DPA)	0.85 ± 0.06	0.64 ± 0.19	0.47 ± 0.11	0.08 ± 0.02	0.89 ± 0.14	0.55 ± 0.03
22:6 *n*-3 (DHA)	ND	0.99 ± 0.30	1.10 ± 0.22	1.00 ± 0.17	1.10 ± 0.21	4.42 ± 0.26
**∑HUFA**	**11.24 ± 0.45**	**7.76 ± 1.76**	**5.32 ± 0.77**	**3.33 ± 0.68**	**8.05 ± 0.65**	**8.34 ± 0.52**
**∑Others**	**2.16 ± 0.46**	**1.23 ± 0.20**	**0.81 ± 0.21**	**3.15 ± 1.27**	**3.56 ± 0.24**	**0.04 ± 0.02**
**∑ Total FA**	**41.58 ± 2.74**	**39.78 ± 8.48**	**12.51 ± 1.62**	**25.22 ± 7.04**	**36.19 ± 4.43**	**97.44 ± 11.23**

Average values ± (SD).

The bold values represent the sum (∑) of SFA saturated FA, *MUFA* mono-unsaturated FA, *PUFA* polyunsaturated FA, *HUFA* highly unsaturated FA, Other—FA identified from microbiome and iso and anteiso (Supplementary Table S1) and total FA. AdA adrenic acid, ALA alpha-linolenic acid, ARA arachidonic acid, DHA docosahexaenoic acid, DPA docosapentaenoic acid, EPA eicosapentaenoic acid, LA linoleic acid. ND—FA not detected. PUFA defined as all FA with ≥ 2 double bonds and HUFA all FA with ≥ 4 double bonds (not considered within ∑PUFA).

**Table 2:** SIMPER overall average dissimilarities (%) between fatty acids (FA) profile of wild and cultured polychaete *Hediste diversicolor*.

*H. diversicolor*		
Wild and IMTA-cultured		
FA	Contrib.%	Cum.%
18:3 *n*-3 (ALA)	11.90	11.90
20:4 *n*-6 (ARA)	11.48	23.38
22:4 *n*-6 (AdA)	10.61	33.99
18:2 *n*-6 (LA)	8.56	42.55
22:6 *n*-3 (DHA)	7.69	50.24

*AdA* adrenic acid, *ALA* alpha-linolenic acid, *ARA* arachidonic acid, *DHA* docosahexaenoic acid, *LA* linoleic acid.

### Comparison of fatty acid profiles of different IMTA-cultured polychaete species

**Figure 2:**
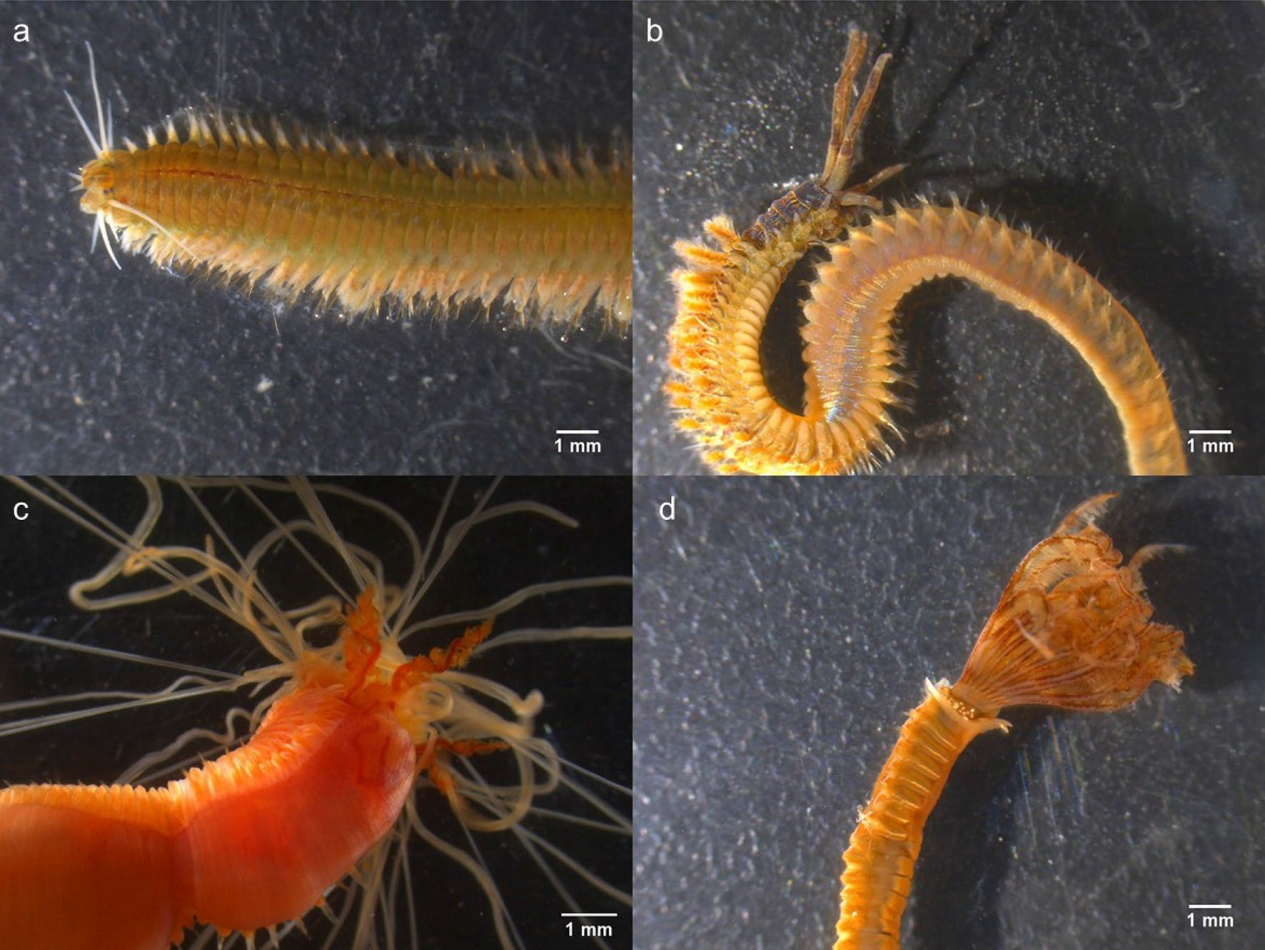
Polychaete species surveyed during the present study: (**a**) *Hediste diversicolor*; (**b**) *Diopatra neapolitana*; (**c**) *Sabella* cf*. pavonina* and (**d**) *Terebella lapidaria*.

**Figure 3:**
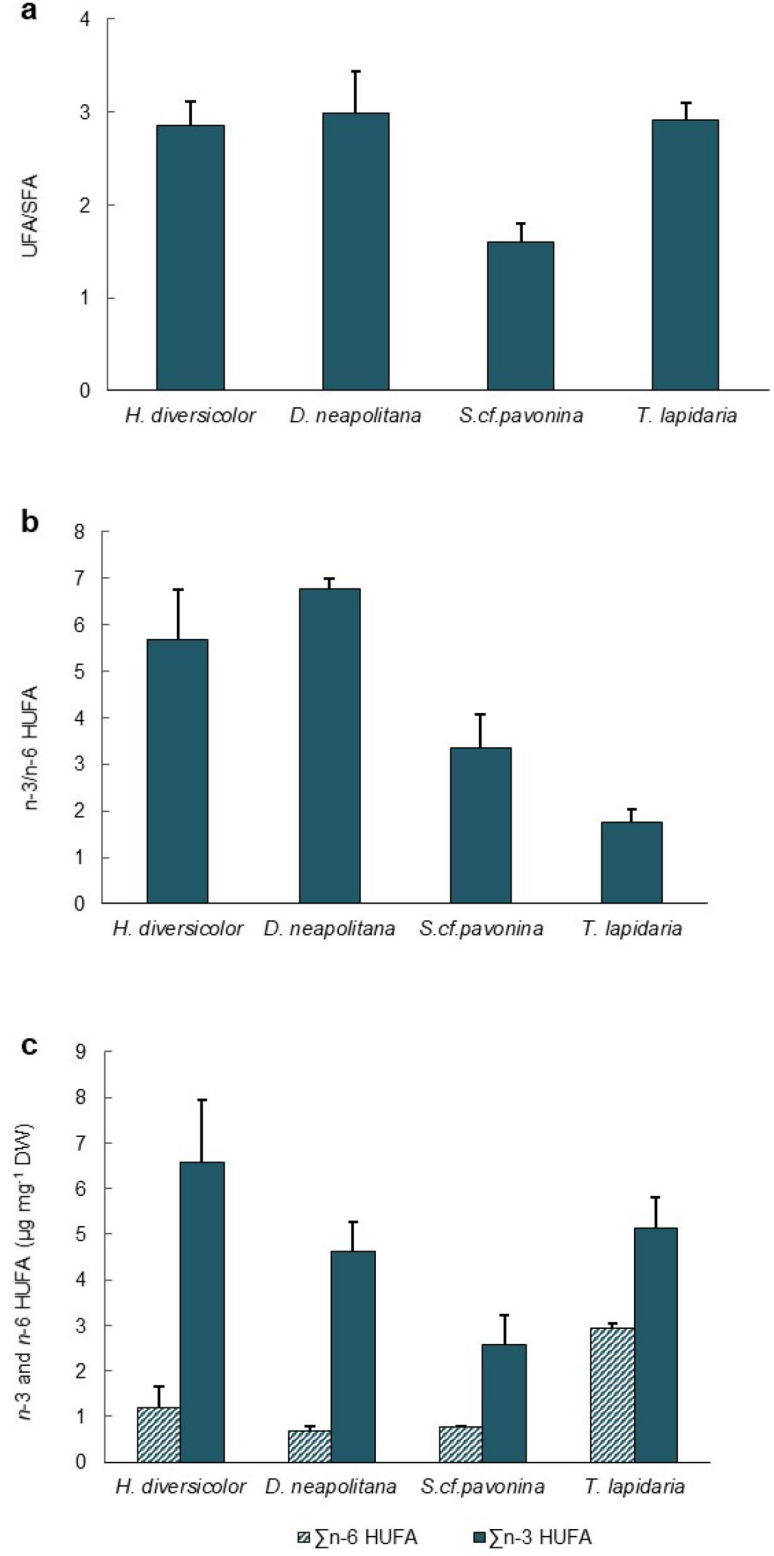
Fatty acid profile of different IMTA-cultured polychaetes (*Hediste diversicolor, Diopatra neapolitana, Sabella* cf. *pavonina* and *Terebella lapidaria*): (**a**) unsaturated and saturated fatty acids ratio (UFA/SFA); (**b**) *n*-3/*n*-6 highly unsaturated fatty acids ratio (*n*-3/*n*-6 HUFA); (**c**) sum of *n*-3 and *n*-6 highly unsaturated fatty acids content (∑n-3 and n-6 HUFA; values in µg mg^−1^ DW). Average values ± SD (n = 5).

The FA profile of IMTA-cultured polychaetes *H. diversicolor*, *D. neapolitana*, *S.* cf. *pavonina* and *T. lapidaria* (Fig. 2) are summarized in Table 1. Apart from *H. diversicolor*, the FA profile of all other polychaete species is here described for the first time. A total of 22, 25 and 28 FA (excluding FA from microbiome and iso and anteiso—Supplementary Table S1) were identified for *D. neapolitana*, *S.* cf. *pavonina and T. lapidaria*, respectively. Significant differences were found between the FA profiles of the four IMTA-cultured polychaete species (ANOSIM test; R = 0.968; *p* = 0.001), with SIMPER analysis at a cut-off of 50% revealing the FA that most contributed to dissimilarities between them (Table 3). *Terebella lapidaria* exhibited the FA profile with the lowest dissimilarity for *H. diversicolor*, followed by *S.* cf. *pavonina and D. neapolitana* (Table 3). The polychaetes *H. diversicolor* and *T. lapidaria* exhibited the highest concentration of total FA per mg DW biomass. Palmitic (16:0), sum of oleic and vaccenic (18:1 *n*-9 and *n*-7), LA (18:2 *n*-6) and EPA (20:5 *n*-3) were the SFA, MUFA, PUFA and HUFA (respectively) that revealed the highest content for both polychaete species. The majority of these FA were also the ones most abundant for the other two polychaete species, except stearic (18:0) and 7,13-docosadienoate (22:2 *n*-9) which were the most abundant SFA and PUFA in the FA profile of *D. neapolitana*, and 5,13-docosadienoate (22:2 *n*-9) which was the most abundant PUFA in the FA profile of *S.* cf. *pavonina*. The concentration of DHA (22:6 *n*-3) was similar between the four IMTA-cultured polychaete species (0.99–1.10 µg mg^−1^ DW). *Hediste diversicolor*, *D. neapolitana* and *T. lapidaria* exhibited similar and higher UFA/SFA ratios (Fig. 3a). When analysing the HUFA profile, it was possible to verify that *H. diversicolor* and *D. neapolitana* exhibited the highest *n*-3/*n*-6 HUFA ratio (Fig. 3b), while the highest *n*-3 and *n*-6 HUFA contents were determined in *H. diversicolor* and *T. lapidaria* biomass (Fig. 3c).

**Table 3:** SIMPER overall average dissimilarities (%) between fatty acid (FA) profile of different polychaete species cultured in sand beds using an open integrated multi-trophic aquaculture (IMTA) approach.

*H. diversicolor* and *D. neapolitana*			*H. diversicolor* and *S.* cf. *pavonina*			*H. diversicolor* and *T. lapidaria*		
Avg. dissimilarity: 40.8%			Avg. dissimilarity: 36.5%			Avg. dissimilarity: 15.3%		
FA	Contrib. %	Cum. %	FA	Contrib. %	Cum. %	FA	Contrib. %	Cum. %
18:1 *n*-9 & n-7	16.01	16.01	18:2 *n*-6 (LA)	9.28	9.28	22:4 *n*-6 (AdA)	10.14	10.14
18:2 *n*-6 (LA)	12.90	28.92	20:1 *n*-13 & *n*-11	9.18	18.47	18:1 *n*-14	7.30	17.44
16:0C	12.44	41.36	^Δ5,13^ 22:2 *n*-9^**Sp**^	8.51	26.97	18:2 *n*-6 (LA)	7.10	24.55
18:1 *n*-14^**Hd**^	8.12	49.48	20:5 *n*-3 (EPA)	8.09	35.07	20:5 *n*-3 (EPA)	7.00	31.54
20:1 *n*-13 & *n*-11	7.26	56.74	18:1 *n*-14^**Hd**^	7.84	42.91	20:2 *n*-9 ^Δ5,11^	5.57	37.12
			^Δ7,13^ 22:2 *n*-9^**Hd**^	5.03	47.94	20:4 *n*-6 (ARA)	5.07	42.19
			20:2 *n*-9	4.89	52.84	18:1 *n*-9 & n-7	4.96	47.15

*D. neapolitana* and *S.* cf. *pavonina*			*D. neapolitana* and *T. lapidaria*			*T. lapidaria* and *S.* cf. *pavonina*		
Avg. dissimilarity: 43.2%			Avg. dissimilarity: 39.7%			Avg. dissimilarity: 35.8%		
FA	Contrib. %	Cum. %	FA	Contrib. %	Cum. %	FA	Contrib. %	Cum. %
18:1 *n*-9 & *n*-7	14.57	14.57	18:1 *n*-9 & *n*-7	16.39	16.39	22:4 *n*-6 (AdA)	9.06	9.06
^Δ5,13^ 22:2 *n*-9^**Sp**^	10.72	25.29	16:0C	12.14	28.53	^Δ5,13^ 22:2 *n*-9^**Sp**^	9.01	18.08
16:0C	10.55	35.83	18:2 *n*-6 (LA)	10.10	38.63	20:1 *n*-13 + *n*-11	8.83	26.90
^Δ7,13^ 22:2 *n*-9^**Dn**^	8.06	43.90	22:4 *n*-6 (AdA)	7.92	46.54	18:2 *n*-6 (LA)	6.25	33.15
20:5 *n*-3 (EPA)	6.16	50.05	20:1 *n*-13 & *n*-11	6.86	53.40	20:2 *n*-9	5.84	38.99
						22:5 *n*-3 (DPA)	5.52	44.52
						20:5 *n*-3 (EPA)	5.00	49.52
						18:1 *n*-14	4.57	54.09

The FA identified with bold superscript: Hd, Dn, Tl and Sp were only identified in the species *Hediste diversicolor*, *Diopatra neapolitana*, *Terebella lapidaria* and *Sabella* cf. *pavonina*. respectively.

*AdA* adrenic acid, *ARA* arachidonic acid, *DPA* docosapentaenoic acid, *EPA* eicosapentaenoic acid, *LA* linoleic acid.

### Comparison of fatty acid profiles of IMTA-cultured polychaete species and the aquafeed provided to fish in earthen ponds

The FA content of the aquafeed provided to the fish is detailed in Table 1. Palmitic acid (16:0), along with the sum of oleic and vaccenic acid (18:1 *n*-9 and *n*-7), LA (18:2 *n*-6) and DHA (22:6 *n*-3) were the SFA, MUFA, PUFA and HUFA (respectively) that exhibited the highest levels in the aquafeed.

**Figure 4:**
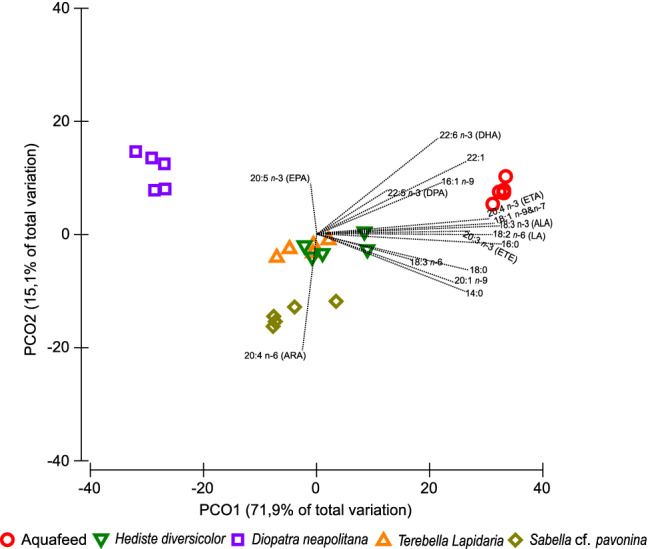
Principal coordinates analysis (PCO) of common fatty acids present in the aquafeed supplied to fish being farmed and the four IMTA-cultured polychaetes (*Hediste diversicolor*, *Diopatra neapolitana*, *Sabella* cf. *pavonina* and *Terebella lapidaria*) (common with at least one of the species). Average values (± SD) (n = 5). *ALA* alpha-linolenic acid, *ARA* arachidonic acid, *DHA* docosahexaenoic acid, *DPA* docosapentaenoic acid, *EPA* eicosapentaenoic acid, *ETA* eicosatetraenoic acid, *ETE* eicosatrienoic acid, *LA* linoleic acid.

The most represented UFA class in the aquafeed was MUFA (≈ 60.5% of all UFA), while in polychaetes the sum of PUFA and HUFA accounted for most UFA (53.2% for *H. diversicolor*, 83.5% for *D. neapolitana*, 51.4% for *S.* cf. *pavonina and* 54.3% for *T. lapidaria*). The FA profile of the aquafeed exhibited a content of *n*-3 HUFA (7.94 ± 0.49 µg mg^−1^ DW) similar to the one reported for *H. diversicolor* and, to a lesser extent, to the one reported for *T. lapidaria.* The levels of DHA (22:6 *n*-3) in the aquafeed per DW biomass was approximately 4-times higher than that recorded in all IMTA-cultured polychaete species. The EPA (20:5 *n*-3) content of all polychaete species, except *S.* cf. *pavonina*, was higher than the one present in the aquafeed. The principal coordinates analysis (PCO) revealed that the FA profiles that more closely resembled that of the aquafeed supplied to the fish being farm in earthen ponds were those of *H. diversicolor* and *T. lapidaria* (Fig. 4). The FA profile of *D. neapolitana* was the less similar to the aquafeed. The two PCO axis explained more than 87% of the variation recorded between samples from different groups.

## Discussion

The current scarcity of new sources of *n*-3 HUFA (mainly EPA and DHA) makes paramount the search for new ingredients from where these EFA can be derived^5^. Polychaetes are likely in the frontline of alternative sources of EFA that can be explored, namely through their integration in IMTA systems as extractive species to recover these valuable nutrients^6, 23, 25, 26, 40^.

*Hediste diversicolor* is well represented in multiple IMTA designs that have already featured its potential to recover nutrients from organic rich effluents^6, 15, 23–28^. The biomass of *H. diversicolor*, whose FA profile was evaluated in present work, was cultured in PASF installed to filter the effluent water of earthen ponds stocked with gilthead seabream (*S. aurata*) during 15 weeks^27^. From an initial inoculum of approximately 400 ind. m^−2^ a density of approximately 1000 ind. m^−2^ (2.5-fold increase) was achieved, with PASF contributing to retain with high efficiency the POM present in the aquaculture effluent (approx. 1.8 ± 1 mg L^−1^)^27^. The significant differences recorded between the FA profile of IMTA-cultured and wild *H. diversicolor* (with an overall dissimilarity of 24.2%) were mainly due to shifts in the concentration of common FA (e.g., ALA [18:3 *n*-3], ARA [20:4 *n*-6], AdA [22:4 *n*-6] and LA [18:2 *n*-6]). This dissimilarity was also due to the presence of less common FA, such as 7,10,13-hexadecatrienoic (16:3 *n*-3) and gamma-linolenic (18:3 *n*-6) which were only identified in wild polychaetes, and DHA (22:6 *n*-3) which was only identified in cultured polychaetes. In general, a total of 35 and 34 FA were identified in wild and IMTA-cultured *H. diversicolor* (respectively) (27 and 26 if FA from microbiome, *Iso* and *anteiso* are excluded). In the present study, it was not possible to conclude that the culture conditions benefit the enrichment of FA profile if evaluated only in terms of total FA content, as the values recorded for wild and IMTA-cultured polychaetes was very similar (≈ 41.6 and 39.8 µg mg^−1^ DW, respectively). Comparing the results recorded in the present study with previous ones which have characterised the FA profile of IMTA-cultured and wild *H. diversicolor* (Table 4), it is possible to verify that total FA content was slightly higher to that displayed by polychaetes supplied with the effluent water of a super-intensive farm of *S. senegalensis* (≈ 37.6 µg mg^−1^ DW)^23^, as well as that recorded for conspecifics supplied with processed water from a *S. aurata* RAS (27.1 µg mg^−1^ DW)^6^. On the other side, Wang et al*.*^25^ reported a slightly higher FA content (56.9 µg mg^−1^ DW) in polychaetes filtering the effluent water from a salmon smolt facility. Conversely to our results, these studies reported increases between 30 and 50% in total FA content of cultured organisms in respect to wild conspecifics (24.4, 17.8 and 41.6 µg mg^−1^ DW, respectively). Pajand et al*.*^24^ also reported a higher FA content (109.9 mg g^−1^ DW) for *H. diversicolor* that filtered the effluent water of beluga sturgeon (*Huso huso*)*,* although no comparison was performed with the FA profile of wild conspecifics. Different size classes of *H. diversicolor* can present different FA profiles (< 30 mm: ≈ 25.4, 30–50 mm: 27.3 and > 50 mm: ≈ 37.6 µg mg^−1^ DW)^15^. In the present study the FA characterisation was performed in specimens with a size > 40 mm and differences recorded with the above-mentioned studies could also be due to different maturation stages and not solely a consequence of contrasting culture conditions (environmental and effluent water nutrient load). The higher concentration of MUFA detected in IMTA-cultured biomass, may be likely a consequence of the FA profile exhibited by the main source of nutrients present in effluent water (the aquafeed provided to *S. aurata*). Pajand et al*.*^24^ obtained a similar result with MUFA and HUFA being the most and least represented FA classes, respectively, in *H. diversicolor* (≈ 39.4% and 4.6% of total FA, respectively) reflecting the formulation of the aquafeed supplied to *H. huso* (≈ 40.5% and 0.6% of total FA, respectively) (Table 4). Bischoff et al.^6^ and Marques et al*.*^23^ verified that HUFA was the major FA class in IMTA cultured polychaetes (≈ 34% and 37.8% of total FA, respectively) when aquafeeds being supplied to fish displayed a higher proportion of HUFA (24% and 20–28% of total FA, respectively) (Table 4). From *H. diversicolor* production under the culture conditions tested in the present work, it can be predicted a generation of approximately 39.8 g of total FA per kg DW biomass, of which ≈ 6.6 g corresponded to *n*-3 HUFA (≈ 4.8 g EPA and 1.0 g DHA). The levels of EPA and DHA measured in IMTA-cultured specimens in the present study differed from the values reported by Marques et al.^23^, as well as those reported by Pajand et al.^24^ (Table 4). These differences likely reflect different culture conditions, mainly the intensification of fish culture conditions and different aquafeeds formulation. In the present work, IMTA-cultured polychaetes displayed EPA (20:5 *n*-3), DHA (22:6 *n*-3), ALA (18:3 *n*-3) and ARA (20:4 *n*-6), with only DHA not being detected in wild conspecifics. Marques et al*.*^23^ did not detect ALA in IMTA-cultured specimens, nor DHA in wild *H. diversicolor*. Bischoff et al.^6^ reported that wild specimens did not exhibit any detectable levels of DHA, ALA and ARA. These finds are likely explained by the seasonal shifts in the lipid content and FA profile that *H. diversicolor* is known to display, with maximum level of lipid content being detected in the winter (19.3% DW) and the lowest during the summer (6.6% DW)^34^.

**Table 4:** Summary of the results of FA characterisation obtained in studies where the species *H. diversicolor* where included in IMTA designs*.*

FA class	Absolute values (µg mg^−1^ DW biomass)							Relative values (% FAMEs)							
	Marques et al*.*^23^					Pajand et al.^24^		Yousefi-Garakouei et al.^26^		Wang et al.^25^			Bischoff et al.^6^		
	Hd (wild)	Hd (SsW)	Fish W	Fish feed A	Fish feed B	Hd (Hh W)	Fish feed	Hd (Om W)	Fish feed	Hd (wild)	Hd (Ssm W)	Fish W	Hd (wild)	Hd (Sa W)	Fish feed
*Nº FA identified*	19	19	18	17	17	19	20	16	17	19	20	18	10	14	11
SFA	6.5	9.00	13.2	18.8	36.5	(26.9)	(57.4)	34.0	22.9	29.4	29.5	40.9	(36.0)	(34.0)	(36.0)
MUFA	6.7	10.1	14.2	37.5	28.8	(43.29	(62.5)	24.8	31.5	24.4	25.4	36.9	(24.0)	(23.0)	(26.0)
PUFA	2.3	4.0	5.3	14.5	10.8	(34.7)	(34.46)	(33.6)	(38.8)	(14.0)	(13.9)	(10.1)	(1.0)	(9.0)	(14.0)
HUFA	8.8	14.2	2.5	17.5	29.5	(5.1)	(0.97)	(7.1)	(6.8)	(32.2)	(31.2)	(12.3)	(39.0)	(34.0)	(24.0)
*20:5 n-3 (EPA)*	5.5	8.3	0.1	7.1	16.2	3.4	0.3	5.6	2.8	22.8	19.1	0.6	(39.0)	(24.0)	(11.4)
*22:6 n-3 (DHA)*	ND	0.8	1.7	8.3	10.6	1.6	0.6	2.1	4.0	1.4	5.4	6.2	–	(4.0)	(13.0)
*n-3 HUFA*	(7.5)	(10.9)	(2.1	(16.6)	(28.1)	(5.1)	(1.0)	(7.7)	(6.8)	(28.2)	(27.9)	(11.8)	(40)	(32)	(25)
*n-6 HUFA*	(1.2)	(3.4)	(0.5)	(0.9)	(1.4)	ND	ND	ND	ND	4.1	3.3	0.5	ND	(6)	(ND)
*n-3*/*n-6 HUFA*	(6.3)	(3.2)	(4.2)	(18.4)	(20.0)	–	–	–	–	(6.9)	(8.5)	(23.6)	–	(5)	–
Total FA (µg mg^−1^ DW)	(24.4)	(37.6)	(35.3)	(88.3)	(105.6)	(109.9)	(155.3)	–	–	41.6	56.9	47.2	17.8	27.1	24.5
Total lipid (mg g^−1^ DW)	–	–	–	–	–	–	-	*-*	*-*	125.5	123.6	–	–	–	–
Total lipid (% DW)	–	–	–	–	–	11.6	20.4	22.2	15.6	–	–	–	–	–	–
Total protein (%DW)	–	–	–	–	–	49.3	41.8	59.7	41.5	–	–	–	–	–	–

Table summarizes the FA characterisations of wild and IMTA-cultured *Hediste diversicolor (Hd)* depending on the origin of wasted nutrients: SsW—*Solea senegalensis* waste; Hh W—*Huso huso* waste; Om W—*Onchorhynchus mykiss* waste; Ssm W—salmon smolt waste; Sa W—*Sparus aurata* waste. Other FA characterisations corresponded to fish W (waste—faeces and uneaten feed) and fish feed. PUFA defined as all FA with ≥ 2 double bonds and HUFA all FA with ≥ 4 double bonds (not considered within ∑PUFA). The values between brackets were estimated based on the FA profile reported in each work.

In this study it was also possible to compare the FA profile of *H.diversicolor* with that of other polychaete species (*D. neapolitana*, *S.* cf. *pavonina* and *T. lapidaria*) which adapted to the conditions in PASF and were identified as potential candidates to integrate IMTA designs as extractive species^27^. The planktonic larvae of the three polychaete species mentioned above successfully colonized the sand beds of PASF, most likely because the substrate of these filters provided the specific cues required for their larvae to settle and metamorphose (e.g., free FA have been suggested to favour the settlement of some species^41^). To date, the performance of *D. neapolitana* had never been tested under an IMTA framework. The adults of this species can present sizes ranging between 150 and 500 mm in length, being one of the species most intensively harvested in the coastal lagoon where the present study was performed^42^. This polychaete reveals an iteroparous reproduction behaviour with a discontinuous reproductive season (spawning: March–July; resting: August–September)^43^. From the four polychaete species whose FA profiles were evaluated in the present work, it was *D. neapolitana* that exhibited the lowest content of total FA with the *n*-3/*n*-6 HUFA ratio being similar to that of *H. diversicolor*. Despite this similarity, in overall, *D. neapolitana* was the species whose FA profile showed a greater dissimilarity to that of *H. diversicolor*. An analysis of *D. neapolitana* productivity in terms of FA profile allowed us to estimate the generation of approximately 12.5 g of total FA per Kg DW biomass produced, of which approximately 40% corresponded to *n*-3 HUFA (including EPA and DHA). As the FA profile of wild specimens of *D. neapolitana* has never been determined, it is impossible to verify if IMTA conditions enhance their value in EFA. Previous studies showed that this species reveals a lower capacity to grow in highly organic enriched areas^44^, a fact that may constraint its use in more intensive IMTA systems. The development of sustainable production models for *D. neapolitana* is paramount^43^, as the level of exploitation (eventually even overexploitation) and inherent digging activity may result in an enhanced biodiversity loss in the benthos^42^. In terms of bioremediation, it must be highlighted that these organisms are ecosystem engineers that stabilise the sediment with the tubes they secrete and thus increase the structural complexity and biodiversity of their habitat^43, 45, 46^, a feature that may contribute for a less pronounced bioturbation. For this reason, this species is likely less suitable to promote safeguard the functionality of PASF tested in present work, as these required complete percolation of water through the substrate^27^.

The species *S.* cf. *pavonina* inhabits the tubes that the worm secretes, and it feeds by using crowns of ciliated filaments on their heads^47^. This polychaete can achieve a size of 270 mm, with an additional 45 mm of its branchial crown^48, 49^. It displays a filter feeding behaviour and is a gonochoristic broadcaster that displays an annual reproductive cycle (spawning period in May/June)^49^. There is no evidence of this species having ever been included in IMTA designs as an extractive species. The total FA content detected in this polychaete species was lower than that of *H. diversicolor*, being also the species which exhibited the lowest *n*-3/*n*-6 HUFA ratio. *Sabella* cf. *pavonina* exhibited a FA profile slightly more similar to that of *H. diversicolor* than the one observed for *D. neapolitana*. An analysis of *S.* cf. *pavonina* productivity in terms of FA profile allowed us to estimate the generation of approximately 25.2 g of total FA per Kg DW biomass produced, of which approximately 10% corresponded to *n*-3 HUFA (including EPA and DHA). It is known that this species can filter more than 70 L of seawater per hour^50^. However, no major enhancement of bioturbation in PASF could be perceived for this tubiculous polychaete, which, like *D. neapolitana* and for the same reasons, does not appear to be a species indicated to promote the functionality of PASF.

Concerning the polychaete *T. lapidaria*, this species can achieve a size of 100 mm^51^ and is characterized by the presence of a feed collecting apparatus formed by numerous tentacles that secrete mucus to trap different feed items^52^. Until the present study, it has never been considered for culture or tested using IMTA conditions. The high culture density recorded in the present study at the end of experimental period (> 4000 ind. m^−2^) revealed the great potential that this worm presents to adapt to these systems^27^. The results here reported are the first FA characterization for *T. lapidaria.* This polychaete species exhibited the most similar FA profile to *H. diversicolor*, concerning total FA content and FA composition. Despite having a concentration of *n*-3 HUFA similar to *H. diversicolor* and *D. neapolitana*, this species exhibited the lowest *n*-3/*n*-6 HUFA ratio, due to the fact that it has a concentration of *n*-6 HUFA higher than all other polychaete species tested in the present work. An analysis of *T. lapidaria* productivity in terms of FA profile allowed us to estimate the generation of approximately 36.2 g of total FA per Kg DW biomass produced, of which approximately 15% correspond to *n*-3 HUFA (including EPA and DHA).

In the evaluation of which of the four IMTA-cultured polychaete species exhibited the FA profile that most resemble that of aquafeed formula (diet supplied to *S. aurata* produced in earthen ponds) it was concluded that *H. diversicolor* and *T. lapidaria* were the species whose FA profile displayed the highest similarity. These polychaetes revealed the higher contents of *n*-3 and *n*-6 HUFA in their composition. This allowed us to assume that both species featured an EFA profile more suitable to be integrated in premium aquafeeds formulation. Here it is important to bear in mind that the differences in *n*-3 and *n*-6 FA profile exhibited by the different polychaete species may likely be explained by its contrasting abilities to produce FA de novo.

The four polychaete species display different feeding habits and explore different trophic niches. *Hediste diversicolor* is considered a discrete motile polychaete, classified as an active predator^53^. This omnivorous species may exhibit a deposit-feeding behaviour and mainly consumes organic matter from substrate^53, 54^. *Diopatra neapolitana* is considered a discrete motile polychaete, omnivorous, a scavenger and detritus feeder^39, 53, 55^. *Terebella lapidaria* is sessile or a discretely motile polychaete and a surface deposit feeder^53^ that traps detritus, including unicellular algae (e.g., diatoms), and various small invertebrates (including larvae) with the mucus secreted by its tentacles, which transfers feed to its mouth^52, 53^. This species also benefits from sediment enrichment in POM derived from fish production^56^. The polychaete *S.* cf. *pavonina* is a sessile species that display filter feeding behaviour and can feed both on phytoplankton (e.g., pelagic diatoms, dinoflagellates, other unicellular algae), small invertebrates (including larvae) and POM dissolved in water column, thus contributing to making the water clearer^32, 48, 53^. Due to these different feeding habits, the nutrition of the four polychaete species surveyed may be more or less on dependent on the POM fraction derived from uneaten fish feed.

The present work demonstrated that it is possible to produce polychaetes biomass with high nutritional value through an eco-design concept such as IMTA, a framework that promotes a cleaner production and, in this case, allowed to recover EFA commonly wasted in aquaculture effluents. The potential of using *H. diversicolor* to recover nutrients, namely EFA, present in the effluent water of earthen ponds used for finfish aquaculture was confirmed. It was also shown that it is feasible to co-culture several other polychaete species in deep sand beds stocked with *H. diversicolor* through the natural settling of planktonic larvae (e.g., *D. neapolitana*, *S*.cf. *pavonina* and *T. lapidaria*). All species displayed different FA profiles, but all hold the potential to recover available nutrients in the effluent water and give origin to value-added biomass, rich in EFA (namely *n*-3 HUFA, such as EPA and DHA). The FA profile of *D. neapolitana*, *S.* cf. *pavonina* and *T. lapidaria* was described here for the first time demonstrating that it is feasible to diversify the polychaete species to be included in PASF. As polychaetes with planktonic larvae will likely always appear in IMTA designs similar to the ones described in the present work, further studies are necessary to maximize the polyculture potential of marine polychaetes using PASF.

## Material and methods

### Experimental set-up

**Figure 5:**
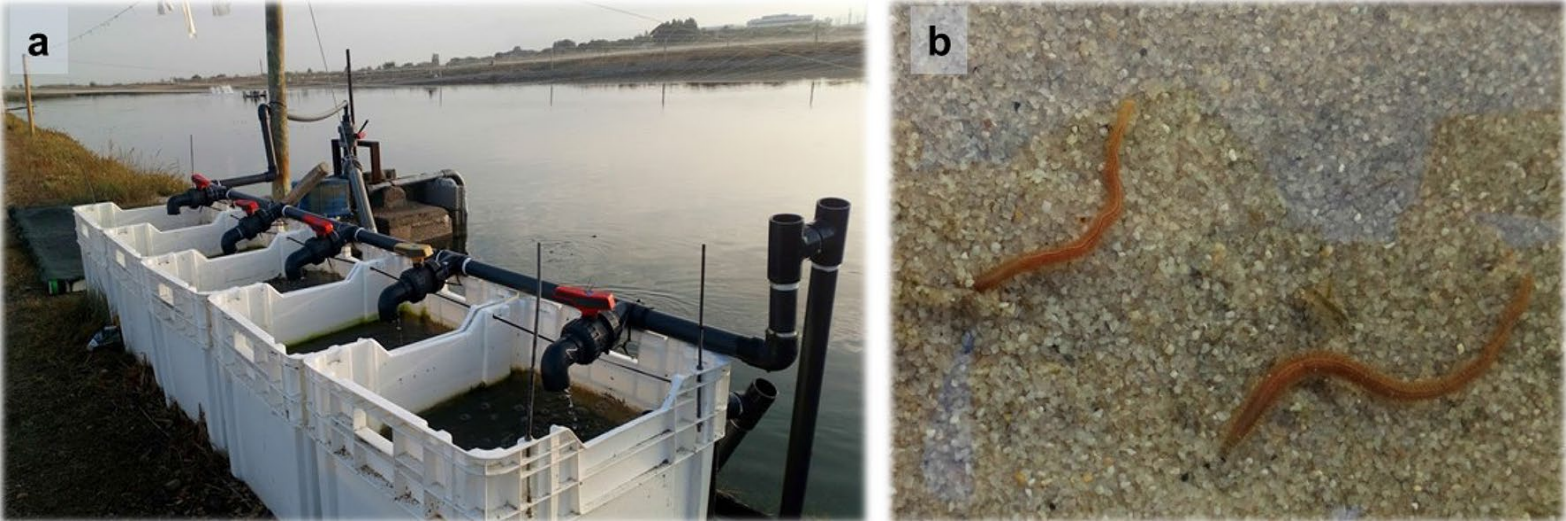
(**a**) Polychaete assisted sand filters (PASF) used in the present study; (**b**) *Hediste diversicolor* in sand bed.

The biomass of polychaetes whose FA profiles were evaluated in present work resulted from an IMTA study performed at AlgaPlus (40° 36′ 43″ N, 8° 40′ 43″ W), an aquaculture company operating in Ria de Aveiro coastal Lagoon watershed area (western Atlantic coast of Portugal)^27^. The present study used the POM fraction of the effluent water from a semi-intensive production pond stocked with gilthead seabream (*Sparus aurata*). Approximately ≈ 12.000 fish with an average weight of 400 g were stocked, being fed twice a day (specific feeding rate ≈ 1.2% day^−1^) on a commercial diet with 43% crude protein, 17% crude fat and 10% crude fibre (Standard orange 4; Sorgal). The effluent water was pumped to 5 tanks arranged in a parallel set-up. Each tank had a volume of 0.1 m^3^, a surface area of 0.3 m^2^ and its bottom was covered by a 200-mm tall sand bed (0.7–1 mm grain size). A 100 mm water column was used on each tank by placing an external standpipe regulating the water level. The standpipe was also connected to a bottom draining pipe that allowed full water percolation through the sand bed. Each tank received a water flow of 25 L h^−1^ (0.5 tank volume renewal h^−1^). An image of the experimental set-up is presented in Fig. 5. The experimental trial was run for 15 weeks, from (July 2017 to November 2017) and no additional feed was supplied to the tanks with the sand bed besides the fish farm effluent. The characterisation of the environmental (Temp., oxygen, pH, salinity) and water composition (SPM, POM, TN, DIN, TP and DIP) conditions of effluent filtered by PASF, as well as the efficiency of bioremediation and productivity achieved in these systems are described in detail in Jerónimo et al.^27^.

### Polychaetes stocking and sampling

Wild specimens of *H. diversicolor* were collected at Ria de Aveiro (40° 47′ 23″ N, 8° 40′ 23″ W) by local fisherman and each of the 5 tanks with a sand bed was inoculated with 440 ind. m^−2^ (≈ 167 g FW m^−2^). As the effluent originated from earthen ponds supplied by the coastal lagoon (Ria de Aveiro) was not pre-filtered, the presence of other polychaete species, namely in their larv planktonic phase was expected to co-occur in the experimental units. At the end of the experimental period, polychaetes were collected with hand core samples (Ø 75 mm, 150 mm depth; N = 5). Specimens were sorted in situ and transported to the laboratory for taxonomic identification while alive and further processing. All specimens were depurated overnight in aerated containers holding pre-combusted sterilized sand and artificial seawater to safeguard empty guts and no potential bias of FA analysis. Following depuration, all polychaetes were freeze-dried and stored at − 80 °C before further analysis.

The FA profiles of *H. diversicolor* stocked in the tanks was also compared with that of other polychaete species whose planktonic larvae successfully settled on the sand beds, namely *Diopatra neapolitana* (Onuphidae), *Sabella* cf. *pavonina* (Sabellidae) and *Terebella lapidaria* (Terebellidae). For each species, a composite sample per tank was used for FA analysis. The same procedure was applied to generate 5 composite samples of wild specimens of *H. diversicolor* from the collection site at Ria de Aveiro. For the species *H. diversicolor*, *D. neapolitana* and *S.* cf*. pavonina* 5 polychaetes were considered for each composite sample, while for *T. lapidaria* 20 polychaetes were considered due to the lower size of their specimens. In addition, 5 samples of fish feed were freeze dried and stored at − 80 °C before FA analysis.

### FA extraction and analysis

The FA content was quantified by screening the fatty acid methyl esters (FAME) obtained through gas chromatography-mass spectrometry (GC–MS) following a well-established method currently on use in our laboratory^57–59^. To prepare the FAME all freeze-dried samples were powdered and homogeneized. Then, 1 mL of *n*-hexane containing 10 µg mL^−1^ of the internal standard C19:0 was added to 10 mg of biomass. Then, 200 µl of methalonic (MeOH) KOH solution (2 M) was added, and the tube was sealed and mixed vigorously in a vortex shaker for 2 min. Following this procedure, 2 ml of saturated NaCl solution (aqueous solution of 1 g NaCl in 100 mL Milli-Q water) was added to the tube, and the mixture was centrifuged for 5 min at 2000 rpm. Following centrifugation, 20 µL of the organic phase was transferred into another tube, was dried under a stream of nitrogen gas and store at − 20 °C until FAME analysis. Immediately before analysis, the FAME were dissolved in 100 µL of hexane and 2 µL of this solution was analysed by gas chromatography–mass spectrometry system (GC–MS) (Agilent Technologies, USA) connected to an Agilent 5973 Network Mass Selective Detector (70 eVand and m/z 50–550 in a 1 s cycle), and equipped with a DB-FFAP column (30 m long, 0.32 mm internal diameter, and 0.25 μm film thickness) (J & W Scientific, Folsom, CA).The oven temperature programmed were as follows: (1) initial temperature of 80 °C for 3 min; (2) linear increase to 160 °C (25 °C min^−1^); (3) linear increase to 210 °C (2 °C min^−1^); (4) linear increase to 250 °C (30 °C min^−1^); (5) standing at 250 °C for 10 min. The temperatures of injector and detector were 220 and 280 °C, respectively. Helium was used as the carrier gas (1.7 mL min^−1^). The FA content of the fish feed was determined, using 1 mL of *n*-hexane containing 0.75 µg mL^−1^ of internal standard (C19:0) added to 15 µg of the lipid extract. All remaining procedures were identical as described above. The FA identification was performed by matching with a previously injected standards mixture (Supelco37 Component FAME Mix, Sigma-Aldrich), as well as by comparing each MS spectrum with a database (AOCS lipid library). The FA content (µg mg^−1^ dry weight, DW) in the samples analysed was calculated based on an external calibration curve using a certified standard mixture (Supelco37 Component FAME Mix, Sigma-Aldrich) and C19:0 as internal standard. The FA 18:4 *n-*3, 22:3 *n*-6, 22:4 *n*-6, 22:5 *n*-3, 22:5 *n*-6, 16:3 *n*-3, 24:2, 13-methyl-14:0 iso, 14-methyl-15:0 iso &13-methyl-15:0 anteiso, 14-methyl-16:0 anteiso, 10-methyl-16:0, 7-methyl-hexadec-6-enoate and 16-methyl-17:0 iso were determined based on the reference values of the FA 18:3 *n*-3, 22:2, 23:0, 22:6 *n*-3, 22:6 *n*-3, 16:0, 24:1 *n*-9, 15:0, 16:0, 17:0, 17:0, 17:0 and18:0, respectively. In the present study, PUFA were defined as all FA with two or more double bounds, while HUFA refers to all FA with four or more double bonds.

### Statistical analysis

Statistical analysis was performed using PRIMER v6 with the PERMANOVA + add-on. In order to ascertain differences in the FA content (µg mg^−1^ DW) of wild and cultured *H. diversicolor*, a 1-way analysis of similarities (ANOSIM) was performed on a resemblance matrix produced using Bray Curtis similarity coefficient of data previously transformed using the formula log (x + 1). A SIMPER analysis was also performed to evaluate which FA contributed the most to the dissimilarities recorded between samples mentioned above until a total of 50% cumulative dissimilarity was achieved. A 1-way ANOSIM and SIMPER analysis using the same criteria was used to highlight the differences in FA profiles between stocked *H. diversicolor* and other polychaete species whose planktonic larvae successfully settled in the experimental units (*D. neapolitana*, *S.* cf. *pavonina* and *T. lapidari*a). To determine which species displayed the FA profile that most closely resembled the FA source (aquafeed provided to fish), resemblance matrixes of the 16 most common FA between feed and polychaetes (common with at least one species) were prepared using Bray Curtis similarity coefficient of the data previously log (x + 1) transformed and then a principal coordinates analysis (PCO) plot was performed.

Those FA known to be related to the microbiome (15:0, 17:0, 17:1 *n*-8 and 17:1 *n*-9) and others (13-methyl-14:0, 14-methyl-15:0 + 13-methyl-15:0, 10-methyl-16:0, 7-methyl-hexadec-6-enoate, 16-methyl-17:0) were not included in the above-mentioned analysis. For a detailed description of all the statistical methods referred employed above please see Anderson et al.^60^.

## Supplementary Information

Supplementary Information.

## Data Availability

All data generated or analysed during this study are included in this published article and its Supplementary Material files.
