# Supplementary material for: Unravelling the fatty acid profiles of different polychaete species cultured under integrated multi-trophic aquaculture (IMTA)
Source: Sci Rep. 2021 May 24;11:10812. doi: 10.1038/s41598-021-90185-8 (PMC8144190; doi:10.1038/s41598-021-90185-8)
Supplement: Supplementary file 1 — Supplementary Information. [file 41598_2021_90185_MOESM1_ESM.docx]

**Supplementary Information for**

**Unravelling the fatty acid profiles of different polychaete species cultured under integrated multi-trophic aquaculture (IMTA)**

Daniel Jerónimo*, Ana Isabel Lillebø, Elisabete Maciel, M. Rosário M. Domingues, Javier Cremades, Ricardo Calado*

*Corresponding authors:

E-mail: [danieljeronimo@ua.pt](mailto:danieljeronimo@ua.pt); Tel.: +351938547866 (D Jerónimo)

E-mail: [rjcalado@ua.pt](mailto:rjcalado@ua.pt); Tel.: +351234370779 (R Calado)

Table S1. Average values (±SD) (n=5) of fatty acid composition of total lipids (µg mg^-1^ DW) identified as others (microbiome and iso and anteiso FA) in wild and IMTA-cultured polychaete species (*Hediste diversicolor*, *Diopatra neapolitana*, *Sabella* cf. *pavonina* and *Terebella lapidaria*,) and aquafeed supplied to farmed fish.

| Fatty acid | *H. diversicolor* (Wild) | *H. diversicolor* | *D. neapolitana* | *S.* cf. *pavonina* | *T. lapidaria* | Aquafeed |
| --- | --- | --- | --- | --- | --- | --- |
| 15:0 | 0.59 ± 0.06 | 0.25 ± 0.05 | 0.08 ± 0.02 | 0.35 ± 0.14 | 0.29 ± 0.02 | ND |
| 17:0 | 1.01 ± 0.30 | 0.55 ± 0.08 | 0.43 ± 0.12 | 0.70 ± 0.35 | 0.75 ± 0.10 | ND |
| 21:0 | ND | ND | ND | ND | ND | 0.04 ± 0.02 |
| 17:1 n-8 ^Δ9^ | 0.08 ± 0.00 | 0.01 ± 0.01 | ND | ND | 0.16 ± 0.01 | ND |
| 17:1 n-9 ^Δ8^ | 0.02 ± 0.02 | 0.07 ± 0.02 | ND | ND | 0.08 ± 0.02 | ND |
| 13-methyl-C14:0 (iso) | 0.18 ± 0.10 | 0.14 ± 0.05 | 0.14 ± 0.05 | 0.43 ± 0.18 | 0.49 ± 0.03 | ND |
| 14-methyl-C15:0 (iso)/ 13-methyl-C15:0 (anteiso) | 0.04 ± 0.03 | 0.04 ± 0.02 | ND | 0.27 ± 0.12 | 0.21 ± 0.02 | ND |
| 14-methyl-C16:0 (anteiso) | 0.12 ± 0.01 | 0.16 ± 0.04 | 0.10 ± 0.02 | 1.07 ± 0.39 | 0.92 ± 0.11 | ND |
| 10-methyl-C16:0 | 0.11 ± 0.01 | 0.01 ± 0.01 | 0.03 ± 0.01 | 0.25 ± 0.12 | 0.21 ± 0.05 | ND |
| 7-methyl-hexadec-6-enoate | ND | ND | 0.04 ± 0.02 | ND | 0.42 ± 0.04 | ND |
| 16-methyl-C17:0 (iso) | ND | ND | ND | 0.08 ± 0.02 | 0.03 ± 0.01 | ND |
| **∑Others** | **2.16 ± 0.46** | **1.23 ± 0.20** | **0.81 ± 0.21** | **3.15 ± 1.27** | **3.56 ± 0.24** | **0.04 ± 0.02** |
